# Protocol for monitoring the growth of *Solanum betaceum* non-embryogenic *callus* using electrochemical impedance spectroscopy

**DOI:** 10.1016/j.xpro.2025.104100

**Published:** 2025-09-19

**Authors:** André Caeiro, Jorge Canhoto, Paulo R.F. Rocha

**Affiliations:** 1 Centre for Functional Ecology, Laboratory Associate TERRA, Department of Life Sciences, University of Coimbra, 3000-456 Coimbra, Portugal

**Keywords:** Cell Biology, Cell culture, Microscopy, Plant sciences, Biotechnology and bioengineering

## Abstract

*Callus* cultures have important biotechnological applications. Here, we describe the use of electrochemical impedance spectroscopy (EIS) as a real-time and non-destructive approach to monitor *callus* growth. We describe the steps to obtain *in vitro* tamarillo clones from seeds and from these non-embryogenic *calluses*. We detail the steps for the preparation of the EIS apparatus and experiment and a parallel growth assay. We also provide complementary microscopy assays to complement the EIS experiment and outline the main data treatment steps.

For complete details on the use and execution of this protocol, please refer to Caeiro et al.^1^

## Highlights

• Instructions for induction of non-embryogenic *callus*
• Protocol for *callus* growth assessment using electrochemical impedance spectroscopy
• Description of complementary microscopy assays
• Guidance on EIS for efficient and non-destructive monitoring of *callus*

## Abstract (editor highlights)

Publisher’s note: Undertaking any experimental protocol requires adherence to local institutional guidelines for laboratory safety and ethics.

## Graphical abstract

**Figure:**
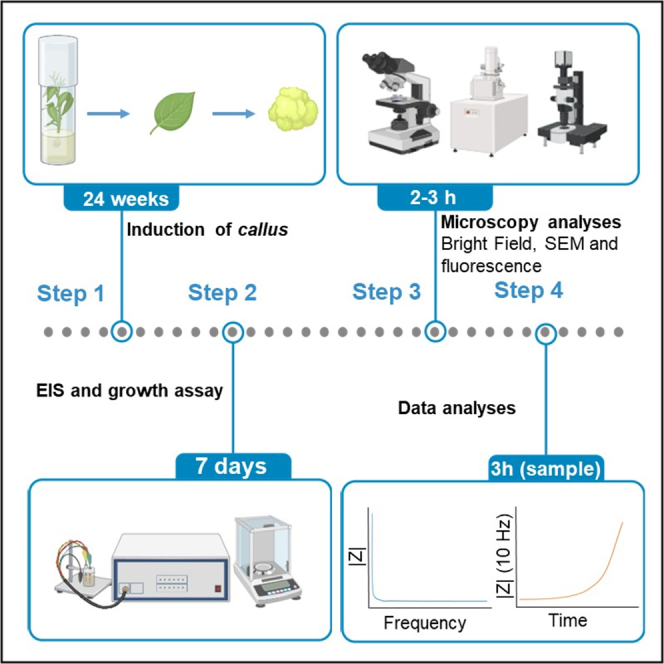

## Before you begin

A methodology to monitor a non-embryogenic *callus* (NEC) from tamarillo cultured leaves obtained and maintained in *in vitro* conditions by Electrochemical Impedance Spectroscopy (EIS) is described.^2^^,^^3^

EIS is an electrochemical screening technique that has been used in several contexts in plant-related fields, namely for root development monitorization, leaf water content and all plant monitorization under controlled conditions (growth chamber).^4^^,^^5^^,^^6^ As an *in vitro* monitoring technique, EIS can have several advantages over classic methodologies such as dry/wet weight and packed cell volume. In fact, EIS is non-invasive and can be employed in real time, which could be a great advantage for continuous biotechnological applications. Furthermore plant cells typically grow on clusters that can increase on volume and biomass, but not on cell number.^7^ EIS by screening the interface of the electrolyte-electrode can potentially detect these changes better than methodologies based on cell number.

The *callus* was induced and assayed in a Murashige and Skoog (MS) medium^8^ supplemented with sucrose and a synthetic auxin (picloram). The induction part of the protocol, which originates NEC, is carried out in leaf segments obtained from clonal red tamarillo lines previously established *in vitro*.^9^ There are several methodologies that can be employed to achieve *in vitro* cloning, such as shoot establishment from adult trees or by using clonal lines derived from seedlings.^9^ In this protocol, we described *in vitro* clones derived from *in vitro* germinated seeds. NEC derived from other initial explants, such as mature zygotic embryos can also be employed.

The protocol employs a two-electrode Electrochemical Impedance Spectroscopy (EIS) system to monitor cellular adhesion and growth. The transducer contains a glass substrate where a pair of circular Au electrodes (6 mm^2^) were thermally evaporated through a shadow mask, on a 10 nm titanium adhesion layer, as previously described.^10^^,^^11^ To validate this methodology, the behavior of NEC in the standard, auxin-rich medium against a formulation without hormone supplementation was tested. Growth assays are made in parallel to validate a correlation between rate of mass increase and rate of increase in impedance modulus. The rate of increase in impedance modulus, at 10 Hz, (|Z|) correlates well with the mass increase of the *callus*. An increase |Z| is correlated to cellular adhesion and growth. The equivalent circuit analysis can also inform on several aspects of cell physiology and be related to cell viability and is, therefore, recommended. Per instance, the charge transfer resistance (*R*_*ct*_), obtained from the equivalent double RC circuit,^2^^,^^3^ differs between the *callus* grown in auxin-free and auxin-rich medium, and this difference is likely related to the different morphology observed in both *callus*.

In this protocol the standard culture medium is MS medium supplemented with picloram (hereafter, referred to as auxin-rich medium). The growth conditions are 24°C under darkness. This is the control condition against which environmental or physiological changes should be measured. Indeed, different medium formulations could potentially be used. If different synthetic auxins or different types of plant hormones are used, the parallel growth assay is required to ascertain the correlation between mass and impedance.

### Innovation

EIS enables continuous, non-invasive and real-time monitoring of *callus* growth. This technique has some advantages over more conventional methods, typically destructive methodologies. Furthermore, because it is sensitive to environmental changes, it can also be used in long-term biotechnological processes, such as continuous biomass production, and to fundamental studies that test *callus* response to external stimuli.

### Stock solutions and culture medium preparation

**Timing: 3–4 h**

1. Prepare solutions of KOH (1 M and 0.1 M).
  a. Dissolve 5.611 g of KOH in 100 mL of distilled water (1 M solution).
  b. Dilute 10 mL of KOH 1 M in 90 mL of distilled water (0.1 M solution).
2. Prepare solutions of HCl (1 M and 0.1 M).
  a. Add 82 mL of HCl 37% to 918 mL of distilled water (1 M solution).
  b. Dilute 10 mL of HCl 1 M in 90 mL of distilled water (0.1 M solution).

**CRITICAL:** Concentrated HCl should be handled in a fume hood with protective gear and extreme caution.

3. Prepare a stock solution of picloram (1 mg/mL).
  a. Dissolve 100 mg of picloram in 1 mL of NaOH 1 M.
  b. Transfer 1 ml of picloram solution to about 30 mL of distilled water under agitation.
  c. Adjust the volume to 100 mL.
  d. Store at −20°C for further use up to a month.
4. Prepare calcium hypochlorite solution (50 g/L).
  a. Dissolve 10 g of calcium hypochlorite in 200 mL of distilled water.
  b. Filter the solution (Whatman paper #1).

***Note:*** The calcium hypochlorite solution should be used immediately.

5. Prepare acetocarmine 1% (w/v) staining solution.
  a. Mix 45 mL of glacial acetic acid in 55 mL of distilled water.
  b. Heat the acetic acid solution in a 1 L Erlenmeyer flash until starts boiling.
  c. Add 1 g of acetocarmine to the acetic acid solution and maintain the temperature at approximately 95°C for 5 min.
  d. Let the solution reach room temperature (20°C).
  e. Filter the solution with a Whatman paper #1 into a dark container.

***Note:*** Acetocarmine solution can be stored for up to 2 years. However, if a precipitate is observed, a filtration step similar to e. should be done before using.

**CRITICAL:** Steps b and c should be performed under a fume hood.

6. Prepare fluorescein diacetate staining solution (2 μg/mL).
  a. Dissolve 100 mg of fluorescein diacetate in 100 mL acetone (stock solution – 1 mg/mL).
  b. Dissolve 9 g of sucrose in 100 ml of distilled water (dilution solution).
  c. Add 20 μL ml of stock solution in 9.8 mL of dilution solution (final volume of 10 mL).

***Note:*** The stock solution can be stored at −20°C for at least 3 months. The dilution and staining solution should be prepared immediately before use.

**CRITICAL:** Acetone is highly flammable and should be handled under a fume hood.

7. Prepare germination medium (half-strength MS medium supplemented with 0.29 mM sucrose).
  a. Dissolve 10 g of sucrose in 800 mL of distilled water.
  b. Add 2.2 g of Murashige and Skoog medium including vitamins to 800 mL of distilled water with sucrose.
  c. Adjust the pH to 5.6–5.8 with KOH (0.1 or 1.0 M) or HCl (0.1 or 1 M) solutions.
  d. Add 7 g of plant agar.
  e. Adjust the volume to 1 L.
  f. Autoclave at 121°C for 20 min.
8. Prepare micropropagation medium (MS medium supplemented with 0.09 M sucrose and 0.88 μM BAP).
  a. Dissolve 30 g of sucrose in 800 mL of distilled water.
  b. Add 4.4 g of Murashige and Skoog medium including vitamins to 800 mL of distilled water with sucrose.
  c. Add 200 μL of BAP stock solution.
  d. Adjust the pH to 5.6–5.8 with KOH or HCl solutions.
  e. Add 7 g of plant agar.
  f. Adjust the volume to 1 L.
  g. Autoclave at 121°C for 20 min.
9. Prepare auxin-rich medium (MS supplemented with 0.26 M sucrose and 20 μM picloram).
  a. Dissolve 90 g of sucrose in 800 mL of distilled water.
  b. Add 4.4 g of Murashige and Skoog medium including vitamins to 800 mL of distilled water with sucrose.
  c. Add 5 ml of picloram stock solution.
  d. Adjust the pH to 5.6-5-8 with KOH or HCl solutions.
  e. Add 2.5 g of Phytagel.
  f. Adjust the volume to 1 L.
  g. Autoclave at 121°C for 20 min.
10. Prepare auxin-free medium (MS supplemented with 26 mM sucrose).
  a. Dissolve 90 g of sucrose in 800 mL of distilled water.
  b. Add 4.4 g of Murashige and Skoog medium including vitamins to 800 mL of distilled water with sucrose.
  c. Adjust the pH to 5.6-5-8 with KOH or HCl solutions.
  d. Add 2.5 g of Phytagel.
  e. Adjust the volume to 1 L.
  f. Autoclave at 121°C for 20 min.

**CRITICAL:** The medium should be distributed to the final container (Petri dishes, microplates or EIS apparatus) immediately after autoclaving at a temperature of about 60°C. Furthermore, if thermolabile compounds are being tested (such as some auxin polar transport inhibitors), the stock solution should be sterilized by filtration (0.2 μm pore filter) and an appropriate volume added at this stage.

### Seed germination and establishment of clonal lines

**Timing: 10–12 weeks**

11. Germinate tamarillo seeds.
  a. Remove the seeds from a mature fruit (any variety can be used).
  b. Wash the fruits in running water for 5 min.
  c. Dry the seeds on Whitman filter paper for 24 h at room temperature (15°C to 20°C).
  d. Add 50 mL each of germination medium to 3 culture boxes (ECO2box/white filter).
  e. Sterilize the seeds by immersing on the hypochlorite solution for 15 min (in this step is advisable to add 2–3 drops of Tween 20).
  f. Wash the seeds with sterilized distilled water 3 times.
  g. Place the seeds in the culture medium (10 to 12 seeds per culture box).
  h. Place the culture boxes in a growth chamber at 24°C in 16 hour photoperiod with a luminous intensity of 25–35 μmol/m^2^.s^1^.

***Note:*** Steps d. to g. must be made under environmental sterility. Complete germination of all viable seeds should occur between 3 and 4 weeks.

12. Establish and multiply clonal lines from the seedlings.
  a. Remove seedlings a seedling from the germination medium to a sterilized Petri dishes using sterilized tweezers.
  b. Using a sterilized scalpel remove the apical portion remove the apical shoot (approximately 1.5 cm).
  c. Place the apical shoot in micropropagation medium with the basal part in contact with the culture medium.
  d. Place the culture boxes in a growth chamber at 24°C with a 16 hour photoperiod at a luminous intensity of 25–35 μmol/m^2^.s^1^.
  e. After 4–5 weeks, remove the clones from the culture medium using sterilized tweezers.
  f. Using sterilized scalpel segment the plantlets at the intermodal region (approximately 1.5 cm).
  g. Place the intermodal regions in micropropagation medium with the basal part in contact with the culture medium.
  h. Place the culture boxes in a growth camber at 24°C with a 16-hour photoperiod at a luminous intensity of 25–35 μmol/m^2^s.^1^
  i. Repeat steps f to h on monthly intervals.

### Induction of non-embryogenic *callus*

**Timing: 12–14 weeks**

**Figure 1:**
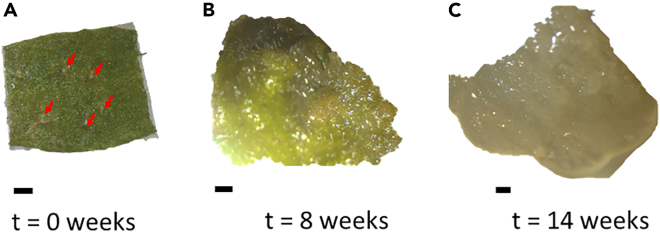
Non-embryogenic *callus* development from leaf segments (A) Initial leaf segment; red arrows point to the punctures. (B) Developing *callus* after 8 weeks. (C) Non-embryogenic *callus.* Bars represent 2 mm.
  e. Place the segments in the Petri dishes with abaxial side in contact with the medium (20 to 25 segments per Petri dish is recommended).
  f. Seal the Petri dishes with Parafilm M.
  g. Place the Petri dishes in a growth chamber at 24°C under dark conditions.
  h. After about 12 weeks, remove the NEC forming on the explants (Figure 1C) and place in fresh medium.
  i. Place the NEC containing Petri dishes in a growth chamber at 24°C under dark conditions.
  j. Subculture NEC as needed by transferring to fresh auxin-rich medium monthly.

13. Induce non-embryogenic *callus* from leaf segments.
  a. Add 30 mL each of the auxin rich medium to 5 Petri dishes (9 cm in diameter and 15.9 mm depth).
  b. Remove the leaves from the tamarillo clones.
  c. Segment the leaves using sterilized scalpel and tweezers (four segments per leaf).
  d. Puncture the segments with the tip of the scalpel 3 to 4 times on the abaxial side (Figure 1A).

**CRITICAL:** All culture and plant manipulation must be made under environmental sterility. Specifically, the tools used to manipulate the plant material must be sterilized before handling using a glass bead sterilizer or similar equipment.

## Key resources table

**Table:** 

REAGENT or RESOURCE	SOURCE	IDENTIFIER
**Biological samples**		

NEC *callus*	Lopes et al.^3^	NA
Tamarillo *in vitro* clones	Correia et al.^5^	NA

**Chemicals, peptides, and recombinant proteins**		

6-benzylaminopurine (BAP)^a^	Sigma-Aldrich	1214-39-7
Acetocarmine^a^	FUJIFILM Wako Pure Chemical Corporation	1390-65-4
Acetone^a^	Sigma-Aldrich	67-64-1
Calcium hypochlorite^a^	Sigma-Aldrich	7778-54-3
Fluorescein diacetate^a^	Sigma-Aldrich	596-09-8
Glacial acetic acid^a^	Sigma-Aldrich	64-19-7
Hydrochloric acid^a^	Sigma-Aldrich	7647-01-0
Isopropanol^a^	Sigma-Aldrich	67-63-0
Murashige and Skoog medium including vitamins	Duchefa Biochemie	M0222
Picloram^a^	Sigma-Aldrich	1918-02-1
Plant agar^a^	Sigma-Aldrich	9002-18-0
Potassium hydroxide^a^	Sigma-Aldrich	1310-58-3
Pythagel^a^	Sigma-Aldrich	71010-52-1
Sucrose^a^	Duchefa Biochemie	57-50-1
Tween 80^a^	Sigma-Aldrich	9005-65-6

**Software and algorithms**		

MATLAB	MathWorks	https://www.mathworks.com/products/matlab.html
ImageJ	Schindelin et al.^12^	https://imagej.org
Zview 2	AMETEK Scientific Instruments	https://www.ameteksi.com/products/software/zview-software-en

**Other**		

Corning Falcon cell culture plate (12 wells)	Sigma-Aldrich	NA
Culture dishes (Petri dishes)	Thermo Fisher Scientific	101IRR
ECO2box/white filter (OVAL model 80 mm H)	Duchefa Biochemie	E1650
EIS setup (transducer holder)	Elghajiji et al.^10^	NA
Glass-gold transducer	Rocha et al.^11^	NA
Whatman qualitative filter paper, grade 1	Merck	WHA1001047
PARAFILM M	Sigma-Aldrich	P7793
Borosilicate glass substrates	CERcuits	https://ceramic-pcb.com/
Solartron SI 1260 impedance analyzer	AMETEK Scientific Instruments	https://www.ameteksi.com/products/software/zview-software-en

a The reagents are identified by the Chemical Abstracts Service (CAS) registry number, a universal identifier. In this case, the source provided is merely indicative of a possible supplier.

## Materials and equipment

**Table:** Acetocarmine 1%, 100 mL

Reagent	Final concentration	Amount
Acetocarmine	1% (w/v)	1 g
Glacial acetic acid	N/A	45 mL g
ddH_2_O	N/A	55 mL

**Table:** Fluorescein staining solution, 10 mL

Reagent	Final concentration	Amount
Fluroescein solution	2 μg/mL	20 μL (stock solution)
Sucrose	0.26 M	0.9 g
ddH_2_O	N/A	10 mL

**Table:** Germination medium, 1 L

Reagent	Final concentration	Amount
Murashige & Skoog (MS) basal medium incl. vitamins)	2.2 g/L	2.2 g
Sucrose	0.29 mM	10 g
Plant agar	0.7% (w/v)	7 g
KOH or NAOH	Adjust pH to 5.6–5.8	N/A
ddH_2_O	N/A	Up to 1 L

**Table:** Micropropagation medium, 1L

Reagent	Final concentration	Amount
Murashige & Skoog (MS) basal medium incl. vitamins)	4.4 g/L	4.4 g
Sucrose	0.09 M	30 g
6-Benzylaminopurine (BAP)	0.88 μM	200 μL (stock solution)
Plant agar	0.7% (w/v)	7 g
KOH or NAOH	Adjust pH to 5.6–5.8	N/A
ddH_2_O	N/A	Up to 1 L

**Table:** Auxin-rich medium, 1L

Reagent	Final concentration	Amount
Murashige & Skoog (MS) basal medium incl. vitamins)	4.4 g/L	4.4 g
Sucrose	0.26 M	90 g
Picloram	20 μM	5 mL (stock solution)
Pythagel	0.25% (w/v)	2.5 g
KOH or NAOH	Adjust pH to 5.6–5.8	N/A
ddH_2_O	N/A	Up to 1 L

**Table:** Auxin-free medium, 1 L

Reagent	Final concentration	Amount
Murashige & Skoog (MS) basal medium incl. vitamins)	4.4 g/L	4.4 g
Sucrose	0.26 M	90 g
Pythagel	0.25% (w/v)	2.5 g
KOH or NAOH	Adjust pH to 5.6–5.8	N/A
ddH_2_O	N/A	Up to 1 L

## Step-by-step method details

### *Callus* monitorization on EIS apparatus and growth assays

**Timing: 7 days per EIS run**

In this step, the monitoring of NEC growth and development is made with EIS. The parallel growth assay allows correlation with the observed variation in |Z| and *R*_*ct*_.

***Note:*** Longer assay periods are possible, however care should be taken to minimize medium evaporation and contamination.

1. EIS apparatus.
  a. Wash the borosilicate glass substrate with acetone sonication for 15 min at room temperature (15°C to 20°C).
  b. Wash the glass substrate with isopropanol sonication for 15 min.
  c. Place an appropriate shadow mask on the glass substrate.
  d. Using a thermal evaporator (for example Edwards 306 thermal evaporator) deposit 10 nm titanium adhesion layer (using for example a Edwards 306 thermal evaporator), followed by the deposition of a 100 nm gold layer.
  e. Remove the transducer from the vacuum chamber of the thermal evaporator and sterilize it by immersing in ethanol (70%) overnight.
  f. Autoclave the EIS apparatus (media holder and electrical connectors) at 121°C for 20 min.
  g. Assemble the EIS apparatus with the sterile Au transducer in a laminar flow hood.
2. EIS Base line measurements.
  a. Add 1 mL of auxin-rich medium to the EIS apparatus.
  b. Wait until the medium jellifies and reaches assay temperature (24°C).
  c. Cover the EIS apparatus with Parafilm M to ensure sterility during the EIS assay.
  d. Start the EIS run in a frequency range from 1 Hz to 1 MHz with AC voltage of 20 mV under dark conditions. Troubleshooting 1.
  e. Collect electrochemical data in 1-hour intervals for 7 days (or the time interval required).
  f. Repeat steps a to e with auxin-free medium.
3. EIS *Callus* measurements.
  a. Add 1 mL of auxin-rich medium to the EIS apparatus with the Au transducer.
  b. Wait until the medium jellifies and reaches assay temperature (24°C).
  c. With sterilized tweezers, take a NEC mass of 20 to 40 mg on top of each circular electrode (the electrodes should be completely covered by the *callus*).
  d. Cover the EIS apparatus with Parafilm M to ensure sterility during the EIS assay.
  e. Start the EIS run in a frequency range from 1 Hz to 1 MHz with AC voltage of 20 mV under dark conditions.
  f. Collect electrochemical data in 1-h intervals for 7 days.
  g. Repeat steps a to f in an auxin-free medium.
4. Mass growth assessment.
  a. Add 1 mL of auxin-rich medium to a well of a 12 Multiple Well Plate.
  b. Wait until the medium jellifies and reaches assay temperature (24°C).
  c. Place a NEC mass of 20 to 40 mg on the well.
  d. Place the plate in a growth chamber at 24°C under dark conditions.
  e. After 24 h, use sterilized tweezers to remove the NEC mass to a pre-weighted sterile Petri-dish sealed with Parafilm M.
  f. Weight the sample.
  g. Return the sample to the same well.
  h. Repeat steps e and f for the duration of the assay.
  i. Repeat steps a to g on auxin-free medium.

***Note:*** EIS measurements (including baselines) and growth assays should be made in, at least, triplicate.

### Microscopy analysis complementary to EIS and growth assays

**Timing: 2–3 h**

In this step, microscopy analysis helps to validate the growth and |Z| changes observed in the previous step and helps the characterization of NEC in terms of morphology and physiology.

5. Bright field microscopy.
  a. Remove a minimal sample of NEC from auxin-rich medium.
  b. Place the sample on a glass slide.
  c. Place 2–3 drops of acetocarmine 1% solution on the glass slide.
  d. Place a coverslip on the sample.
  e. Observe using a Nikon Eclipse Ci microscope (Nikon Instruments Europe BV, Amsterdam, Netherlands) with a Nikon 40x/0.65 NA Plan Achromat objective coupled to a Nikon DS-Fi3 camera (Nikon Instruments).
    ***Note:*** Process the micrographs with the software NIS-Elements D (version 4.60, Nikon Instruments).
  f. Repeat steps a to e in different time points (*e.g.* 3 and 7 days) on samples in auxin-rich and auxin-free medium.
    ***Note:*** Acetocarmine is a general nucleic acid staining agent. To examine specific structures, such as starch granules, different staining agents can be used.
6. Scanning electron microscopy.
  a. Remove a minimal sample of NEC from auxin-rich medium.
  b. Place the sample in a metallic SEM stub sample holder using double-sided conductive carbon tape.
  c. Place the sample holder without further preparation in a variable pressure scanning electron microscope (Flex SEM 1000, Hitachi, Tokyo, Japan) in a low vacuum mode (variable pressure mode).
  d. Set the acquisition parameters as follows: accelerating voltage 15.0 kV, working distance 6.7 mm, signal BSE-COMP, magnification 210 ×, scan speed slow (80), and resolution 1280 × 960 pixels.
  e. Repeat steps a to d on the same time point samples as step 5.
  f. Repeat steps a to e on samples of NEC in auxin-free medium.
7. Viability screening.
  a. Place a sample of about 20 mg of NEC from auxin-rich in a 1.5 mL vial.
  b. Add 1 mL of diluting solution (without fluorescein) - control. Troubleshooting 2.
  c. Vortex for 30 s.
  d. Place 2–3 drops of the suspension in a glass slide.
  e. Cover the suspension with a coverslip.
  f. Place a sample of about 20 mg of NEC from auxin-rich medium in a 1.5 mL vial.
  g. Add 1 mL of fluorescein diacetate (2 μg/mL) solution. Troubleshooting 3.
  h. Vortex for 30.
  i. Incubate for 5 min under dark conditions at 15 to 20°C.
  j. Place 2–3 drops of the suspension in a glass slide.
  k. Cover the suspension with a coverslip.
  l. Repeat steps a to e for a NEC sample in auxin-free medium.
  m. Repeat steps f to j on the same time point samples as step 5.
  n. Observe the slides in an Axio Image.Z2 microscope equipped with a Plan-Apochromat 20×/0.8 NA air objective (M27) coupled to an AxioCam HRm3 monochrome CCD camera.
    ***Note:*** Fluorescence is acquired with a HXP 120 V arc lamp with an excitation and emission wavelength of 450–490 and 500–550 nm, respectively.
  o. Acquire the images using ZEN 3.2 (Blue edition) software at 20× magnification with 300 ms exposure time, 58.3% light intensity and a 14-bit (gray 16) pixel depth.
    ***Note:*** With these settings, the final pixel size is 0.3225 × 0.3225 μm.
  p. Process the images on Fiji software^12^ (1.54f). Use the “auto-adjust” tool for brightness/contrast and measure the raw integrated density of each micrograph.

### EIS data treatment

**Timing: 2–3 h per EIS**

We detail the most important steps to obtain |Z| as a function of time from the raw EIS data.

***Note:*** Care should be taken as different EIS software can present the data in distinct formats.

**Figure 2:**
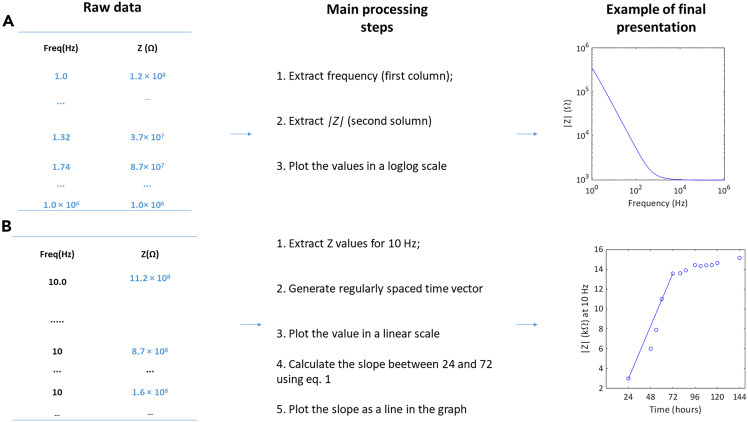
Flowchart of EIS processing steps (A) Analysis of impedance modulus (|Z|) as a function of frequency. (B) Analysis of impedance modulus (|Z|) as a function of time. On the first column the data represented in blue is extracted, the second column shows pseudo-code of the main steps and final column gives an example of the final presentation of the data.
  b. Extract impedance modulus data.
  c. Plot log(Impedance modulus) as a function of log(frequency).
  d. Extract the phase data (given by –tan(Z’’/Z′), where Z″ and Z′ are the imaginary and real part of impedance, respectively.).
  e. Plot Phase as a function of log(frequency).
  f. Repeat steps a to i samples for auxin-free medium.
  g. Repeat steps a to i for NEC samples in auxin-rich and auxin-free medium.

***Note:*** If necessary, other parameters (loss or capacitance) obtained from the EIS experiment can be plotted as a function of frequency using the steps described.

**Figure 3:**
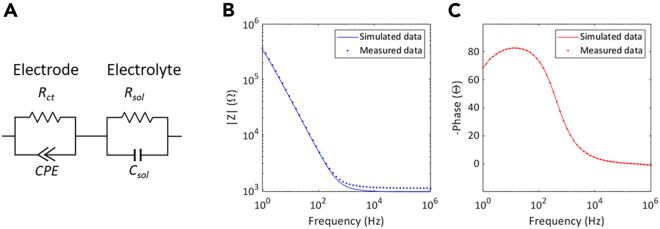
Equivalent circuit analyses (A) Double RC circuit used in modeling. (B) Impedance modulus of as function of frequency. (C) Phase as a function of frequency.
  b. Calculate the *R*_*ct*_ using the double layer capacitance as described in Caeiro et al.^1^
  c. Repeat step a for all samples of auxin-rich medium.
  d. Build a matrix where the columns represent samples and the lines represent the sample repetitions (*e.g.*, *N* × 3, if the assays were made in triplicate).
  e. Determine the arithmetic average for each line.
  f. Determine the standard deviation for each line.
  g. Represent the average values of *R*_*ct*_ as a function of the time interval (determined in step 9f).
  h. Use the standard deviation values for error bars.
  i. Repeat steps a to h for auxin-free medium.
  j. Repeat steps a to h for NEC samples in auxin-rich and auxin-free medium.

8. Plot Impedance, phase and loss data as a function of frequency.
  a. Extract frequency data (Figure 2A).

9. Plot impedance as a function of time.
  a. Extract the values of the impedance modulus at the chosen frequency below the relaxation frequency (in this case, 10 Hz) (|Z|) for auxin-rich medium (obtaining a column of N points) (Figure 2B).
  b. Repeat step a for all samples of auxin-rich medium.
  c. Build a matrix where the columns represent samples and the lines represent the sample repetitions (*e.g.*, *N* × 3, if the assays were made in triplicate).
  d. Determine the arithmetic average for each line.
  e. Determine the standard deviation for each line.
  f. Generate the time interval by creating a vector of evenly spaced values (the interval between points is the time interval between EIS reads, *t*) in the range [0, *N.t*].
  g. Represent the average values of |Z| as a function of the time interval.
  h. Use the standard deviation values for error bars.
  i. Repeat steps a to h for auxin-free medium.
  j. Repeat steps a to h for NEC samples in auxin-rich and auxin-free medium.
10. Plot *R*_*ct*_ as a function of time.
  a. Using an appropriate software (*e.g.*, Zview) model the EIS data of auxin rich medium to the double RC circuit presented (Figure 3A).

## Expected outcomes

In the protocol described the rate of |Z| correlates well with mass increase for both auxin-rich and auxin-free media. The results are particularly relevant in the time interval between 24 and 96 h. The increase in impedance in the first 24 h is associated with *callus* adhesion to the electrode, whereas from 144 h onwards, the impedance stabilizes due to the stationary phase of growth.

**Figure 4:**
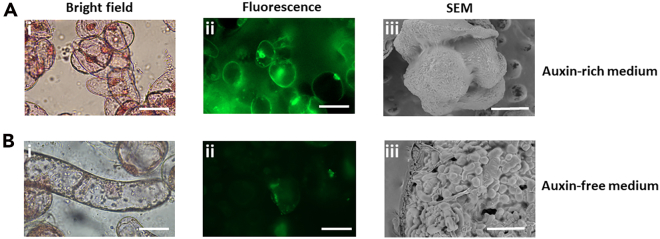
Typical morphologies of the *callus* in the different culture media (A) Auxin-rich medium. (B) Auxin-free medium. The first column represents bight-field microscopy, the second fluorescence (with fluorescein diacetate) and the third scanning electron microscopy (SEM). Bars in the bright-field and fluorescence images represent 50 μm while the bars on the SEM represent 400 μm.

Auxin-rich medium is the standard condition of NEC. Therefore, the morphology and viability of the *callus* should remain unchanged during the 7-day growth period. The typical cellular morphology on bright-field microscopy is shown (Figure 4Ai), with some clustering of oval-elongated cells with a dense nucleus and a high cytoplasmatic volume. The viability of the *callus* as assayed by fluorescein diacetate staining (Figure 4Aii) should present a high number of viable cells (at least 70%) and remain largely unchanged over time. Finally, the cellular hyper structure observed by scanning electron microscopy (Figure 4Aiii) is expected to appear as almost globular with a homogenous surface. On the other hand, if we do not provide auxin to the medium, this presents a detrimental environmental change. In this case, at the end of the experiment (7 days), morphological changes with elongated cytoplasm and nuclear dispersion (Figure 4Bi) are expected, accompanied by a progressive loss of viability (Figure 4Bii). The surface of the *callus* is also expected to change, with a more heterogeneous surface and the appearance of “porous-like” structures (Figure 3Biii). For in depth discussion on the morphological changes on the conditions described see Caeiro *et al.*, 2025.^1^ In fact, at the end of the growth period, NEC in auxin-free medium appears to be entering a stage of necrosis. The rate of mass increase should be minimum. However, in auxin-rich medium, the rate of mass increase should be higher and |Z| should confirm this trend. The higher |Z| increase observed in auxin-rich medium is correlated to the mass increase and can be explained by the greater area covered by the *callus* on the electrode. In fact, in the low frequency analyzed, the electrolyte-electrode interface is being specifically screened so it is expected that a higher mass will lead to a higher opposition to the current and therefore, a higher impedance modulus.

The electrochemical system can be modeled by a double RC circuit (Figure 4A) consisting of a constant phase element (CPE) which represents the double layer capacitance in parallel with a charge transfer resistance *R*_*ct*_. The first RC is essentially in series with a spreading solution which accounts for the electrolyte resistance. The parameters derived from this analysis are discriminative of *callus* general morphology and physiology. *R*_*ct*_, in particular, which is a frequency independent parameter that characterizes the Faradaic redox processes at the electrode-electrolyte interface, should have a distinct profile in auxin-rich and auxin-free medium. It is expected that this parameter is more stable in auxin-free medium with a much lower rate of increase over time. This behavior has been observed and is likely related to the overall morphology changes that occur on the auxin-free medium, specifically the appearance of “pore-like” structures on the surface of the *callus* (Figure 3Biii), which suggests that *R*_*ct*_, in the conditions described, can be used to extract information about the physiological status of the *callus*.^1^ However, more detailed studies are still necessary to clearly explain this behavior. The solution resistance, *R*_*sol*_, which is likely linked to the loss of membrane integrity and subsequent increase in ion concentration, should present a higher rate of increase in auxin-free medium.

Lastly, if different medium formulations are used, *e.g*. supplementation with different auxins or auxin inhibitors, the picloram supplemented medium (auxin-rich medium) should be considered a biological control, and the physiological and morphological observations should be compared to this state. Auxin-free medium, in the conditions described, is a model of early necrosis, and could be used to compare different stages of this process in *callus*, if the added compound is detrimental to NEC.

## Quantification and statistical analysis

The impedance modulus and phase over the full frequency spectrum, from 1 Hz to 1 MHz, is first examined to verify the relaxation frequency of the electrochemical system, which can vary with electrode area, material and solution resistance (see Figures 4B and 4C). After this stage, impedance modulus at 10 Hz (|Z|), before the relaxation frequency,^10^ can be traced as a function of time and related to mass increase. Thus, mass increase should also be represented as a function of time. Both parameters are expected to present an exponential increase phase between 24 h to 72 h (see expected results). In this phase, the rate of increase can be calculated as:

(Equation 1) $$\begin{array}{c} r_{i}=\frac{V_{f}-V_{i}}{t_{f}-t_{i}} \end{array}$$

Where *r*_*i*_ is the rate, *V*_*f*_ and *V*_*i*_ are the |Z| modulus or mass and *t*_*f*_ an *t*_*i*_ are the corresponding times, respectively. The rate will have units of Ω/h or mg/h for |Z| and mass increase, respectively. A similar analysis can be used for *R*_*ct*_ and *R*_*sol*_ when plotted over time.

The goodness of fit of any *r*_*i*_ can be tested using coefficient of determination or a similar metric. Furthermore, mass and impedance values can be normalized between 0 and 1:

(Equation 2) $$\begin{array}{c} X_{n}=\frac{X-X_{\min}}{X_{\max}-X_{\min}} \end{array}$$

Where *X*_*n*_ is the normalized value, *X* is the original data point and *X*_*min*_ and *X*_*max*_ are the minimal and maximum values, respectively. Impedance and mass values can then be analyzed by a correlation test (e.g. Pearson correlation test).

Image processing and treatment can be made in Fiji software.^12^ The area of pores on SEM micrographs can be measured using the appropriate scale tool whereas the fluorescence of the viability assay quantified by the raw integrated density. Analyze the resulting numerical results by standard statistical methods. Firstly, check the homogeneity of variances by the Brown-Forsythe test. If variances are homogenous, use analysis of variance (ANOVA) followed by Tukey’s Multiple comparison test. In the case of non-homogenous variances, use the non-parametric Kruskal-Wallis test followed by Dunn’s multiple comparison.

Finally, equivalent circuit analyses can be performed on appropriate software (such as Zview) using the double RC circuit described previously.

## Limitations

The protocol described applies only to non-embryogenic *callus* on auxin-rich and auxin-free medium. Future studies should compare embryogenic and non-embryogenic *callus*. Validation of this technique in other types of *callus,* particularly from other plants, should be performed. In addition, the analysis is limited to a 2D plane given the planar nature of the electrodes. Detection should be improved by using 3D porous structures, as recently devised.^13^

## Troubleshooting

### Problem 1

|Z| as a function of frequency presents noisy oscillations (Figure 5B).

**Figure 5:**
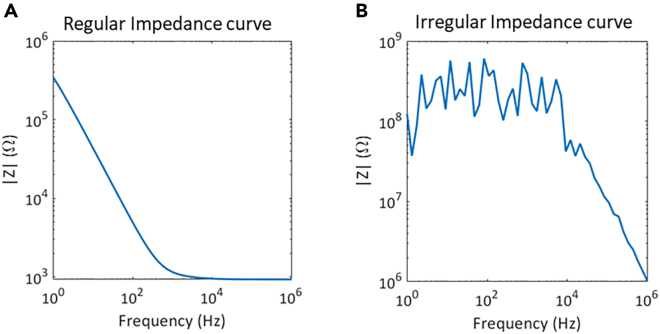
Impedance vs. frequency curves (A) Regular impedance curve. (B) Irregular impedance curve.

### Potential solution

• Check the integrity of the transducer (Au circular electrodes, strip-lines and connection pads).

OR

• Inspect the electrical contacts and cable connections of the system.

OR

• Check for contaminations.

### Problem 2

Sample autofluorescence is high.

### Potential solution

• Reduce the initial amount of NEC mass.

OR.

• Increase the volume of diluting solution.

### Problem 3

Sample fluorescence is too high or too low.

### Potential solution

• Adjust the concentration of fluorescein stain solution (step 2).

## Resource availability

### Lead contact

Further information and resources should be directed to and will be fulfilled by the lead contact, Paulo Roberto Ferreira da Rocha (procha@uc.pt).

### Technical contact

Technical questions on executing this protocol should be directed to and will be answered by the technical contact, Paulo Roberto Ferreira da Rocha (procha@uc.pt).

### Materials availability

This study did not generate new unique reagents.

### Data and code availability

• All the data reported in this paper will be shared by the lead contact upon request.
• This paper does not report original code.

## Acknowledgments

This work was carried out at the R&D Unit Centre for Functional Ecology—Science for People and the Planet (CFE), with reference UIDB/04004/2020, financed by FCT/MCTES through national funds (PIDDAC). P.R.F.R. acknowledges the support and funding from the European Research Council (ERC) under the European Union’s Horizon 2020 research and innovation program (grant agreement no. 947897).

## Author contributions

J.C. and P.R.F.R. contributed to the conception and design of the study and revised the manuscript. A.C. and P.R.F.R. analyzed the data. A.C. performed the experimental work and wrote the manuscript. All authors reviewed and approved the final version of the manuscript.

## Declaration of interests

The authors declare no competing interests.
